# 412. Comparison of Outcomes in Pneumonia Patients Hospitalized with COVID-19 and Influenza in Connecticut, 2015-2020

**DOI:** 10.1093/ofid/ofad500.482

**Published:** 2023-11-27

**Authors:** Daewi Kim, Kimberly Yousey-Hindes

**Affiliations:** Yale CT Emerging Infections Program, New Haven, Connecticut; Yale School of Public Health, New Haven, Connecticut

## Abstract

**Background:**

Since the start of the Coronavirus Disease 2019 (COVID-19) pandemic, comparisons between hospitalized COVID-19 and influenza patients diagnosed with pneumonia are scarce. The objective of this study was to compare characteristics and outcomes among the two.

**Methods:**

Cases for this analysis came from FluSurv-NET and COVID-NET, population-based surveillance platforms of the Connecticut Emerging Infections program for laboratory-confirmed influenza and COVID-19 hospitalizations. FluSurv-NET pneumonia cases occurred during 4 seasons (2015-16 – 2018-19), COVID-NET cases occurred during March-December 2020. Chart reviews were completed for all FluSurv-NET cases and a sample of all COVID-NET cases. Pneumonia was defined as having radiographic key terms on chest x-ray/CT and a discharge diagnosis of pneumonia in the medical record. Outcomes used for comparison included length of stay, intensive care unit (ICU) admission, death, and the following 3 discharge diagnoses: acute respiratory distress syndrome (ARDS), respiratory failure and sepsis.

**Results:**

A total of 1361 pneumonia cases were identified, 652 from COVID-NET (COVID-19 pneumonia[C-19-pna]) and 709 from FluSurv-NET (Influenza pneumonia [I-pna]). C-19-pna cases had a longer median length of stay compared to I-pna cases (9 days vs 5 days; p< .001) and were more likely to have a diagnosis of acute respiratory failure (64% vs 26.3%, p< .001) across all adult age groups (≥18 years). C-19-pna cases were also more likely to die and have an ARDS diagnosis compared to I-pna cases (25% vs 6.1%, p< .001; 10.8% vs 1.7%, p< .001 respectively); this was observed across age groups ≥ 50 years (p < .001). C-19-pna cases ≥65 years were more likely to be admitted to the ICU compared to I-pna cases ≥65 years (35.8% vs 22.2%, respectively p< .001). Conversely, a greater proportion of I-pna cases ≥65 years had a sepsis diagnosis compared to C-19-pna cases in the same age group (24% vs 16.9%, respectively p< .001).

**Figure:**
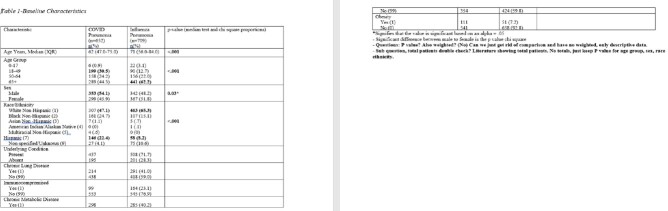

**Figure:**
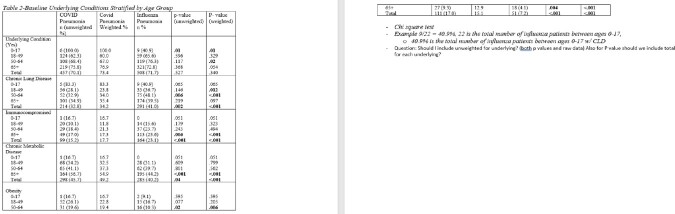

**Figure:**
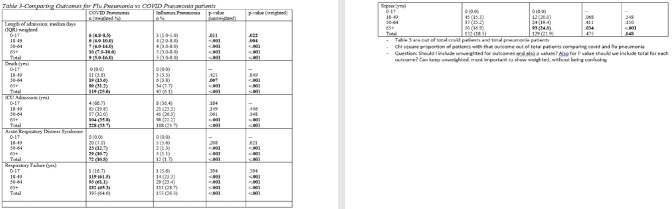

**Conclusion:**

C-19-pna cases were younger, more obese and had more chronic metabolic disease than I-pna cases. I-pna cases were more likely to have chronic lung disease and be immunocompromised than C-19-pna cases. C-19-pna resulted in a significantly longer median length of stay, greater in-hospital mortality, and greater morbidity.

**Disclosures:**

**All Authors**: No reported disclosures

